# Global, regional, and national burden of glucose-6-phosphate dehydrogenase (G6PD) deficiency from 1990 to 2021: a systematic analysis of the global burden of disease study 2021

**DOI:** 10.3389/fgene.2025.1593728

**Published:** 2025-05-23

**Authors:** Zhengyu Yu, Qiang Xiong, Zhongwang Wang, Linfeng Li, Ting Niu

**Affiliations:** ^1^ Department of Hematology, West China Hospital, Sichuan University, Chengdu, China; ^2^ National Center for Occupational Safety and Health, National Health Commission of the People’s Republic of China, Beijing, China

**Keywords:** glucose 6-phosphate dehydrogenase deficiency, global burden of disease, prevalence, years lived with disability, epidemiology

## Abstract

**Background:**

Glucose-6-phosphate dehydrogenase (G6PD) deficiency remains a significant global health burden, particularly in malaria-endemic regions. Despite advances in diagnostic capabilities and treatment strategies, the prevalence and associated disability burden continue to evolve. This study provides a comprehensive assessment of the global, regional, and national burden of G6PD deficiency from 1990 to 2021, using the Global Burden of Disease (GBD) 2021 database.

**Methods:**

Data were retrieved from GBD 2021, covering 204 countries and territories. Prevalence, incidence, and years lived with disability (YLDs) were analyzed. Age-standardized rates (ASRs) and estimated annual percentage change (EAPC) were computed to assess trends over time. The relationship between socio-demographic index (SDI) and G6PD burden was examined using Spearman correlation analysis.

**Results:**

In 2021, global prevalence reached 443,326,869 cases, an 80.17% increase from 1990. The highest burden was observed in South Asia, accounting for 138,159,940 cases. The largest percentage increase in prevalence was in Andean Latin America (+291.96%). G6PD deficiency burden was negatively correlated with SDI, but high SDI regions exhibited higher prevalence than expected. Age- and sex-specific analysis revealed a higher burden in males, particularly in childhood and older age groups.

**Conclusion:**

This study underscores the growing burden of G6PD deficiency, with substantial regional disparities. The findings emphasize the need for improved screening programs, policy interventions, and resource allocation in low- and middle-income countries (LMICs). Continued surveillance is essential to mitigate the long-term health consequences of this disorder.

## Introduction

Glucose-6-phosphate dehydrogenase (G6PD) deficiency is the most common X-linked enzymopathy, affecting approximately 400 million individuals worldwide. This disorder results in impaired redox homeostasis, making erythrocytes highly vulnerable to oxidative stress. Exposure to triggers such as infections, certain medications (e.g., sulfonamides, antimalarials), and dietary oxidants (e.g., fava beans) can precipitate acute hemolytic anemia (AHA) or chronic hemolysis, leading to severe complications including neonatal jaundice and kernicterus (Stykel et al., 2025). The condition remains a major global public health concern, particularly in regions where G6PD deficiency is highly endemic.

The geographical distribution of G6PD deficiency strongly correlates with the historical prevalence of malaria, supporting the hypothesis that heterozygous G6PD mutations confer a selective advantage against Plasmodium falciparum infections (Malaria Genomic Epidemiology Network, 2014). As a result, South Asia, Sub-Saharan Africa, and the Middle East exhibit the highest allele frequencies. However, the burden of G6PD deficiency is no longer confined to malaria-endemic regions due to global migration, increasing population diversity, and improved diagnostic capacity. Advancements in G6PD screening and genetic testing programs in both high-income and low-to-middle-income countries have significantly increased the global recognition and diagnosis of G6PD deficiency, underscoring the necessity for updated surveillance and management strategies worldwide (Taylor et al., 2023).

Despite the well-documented clinical consequences of G6PD deficiency, epidemiological estimates remain inconsistent across regions, with data gaps limiting effective public health planning (Clarke et al., 2017). Previous studies have often relied on hospital-based reports, national health registries, or targeted population studies, which may not fully capture asymptomatic carriers or subclinical cases. The Global Burden of Disease (GBD) 2021 framework provides a standardized approach to estimating prevalence, incidence, and disease burden (years lived with disability, YLDs) across 204 countries and territories, enabling robust comparisons over time and across diverse socio-demographic settings (Naghavi et al., 2024).

The present study aims to provide a comprehensive global analysis of G6PD deficiency from 1990 to 2021, using GBD 2021 data to: (1) Characterize global, regional, and national trends in G6PD deficiency prevalence, incidence, and disability burden; (2) Assess the association between G6PD burden and the Socio-Demographic Index (SDI) to understand disparities across different development levels. By addressing these critical gaps, this study seeks to guide policymakers and clinicians toward targeted prevention strategies and precision medicine approaches in managing G6PD deficiency worldwide.

## Materials and methods

### Data source

This study utilized a subset of the Global Burden of Disease (GBD) 2021 database to investigate the burden of glucose-6-phosphate dehydrogenase (G6PD) deficiency from 1990 to 2021. The GBD 2021 database, curated by the Institute for Health Metrics and Evaluation (IHME) (https://ghdx.healthdata.org/gbd-2021), provides comprehensive disease burden estimates across 204 countries. It encompasses 371 diseases and injuries, 288 causes of mortality, and 88 risk factors spanning the period from 1990 to 2021 (Collaborators G. D. I., 2024; Naghavi et al., 2024; Collaborators G. R. F., 2024). G6PD deficiency was operationally defined in GBD 2021 based on a quantitative reduction in G6PD enzymatic activity. It was primarily assessed via the spectrophotometric assay, recognized as the gold-standard method for quantitative evaluation. Additional assays employed included qualitative fluorescent spot tests and point-of-care rapid screening methods such as the CareStart™ biosensor, ensuring comprehensive coverage and data reliability (Sadhewa et al., 2025; Pal et al., 2019). The burden of years lived with disability (YLDs) was estimated through microsimulation models, incorporating age-, sex-, location-, and year-specific prevalence estimates for each sequela, alongside corresponding disability weights. YLDs were computed by multiplying the number of affected individuals by the duration of disability or time until remission or death, weighted by disability severity (Collaborators G. D. I., 2024).

### Estimation of annual percentage change (EAPC) and percentage change

The temporal trend in age-standardized rates (ASR) of G6PD deficiency between 1990 and 2021 was assessed using a generalized linear regression model. The estimated annual percentage change (EAPC) was derived by modeling the natural logarithm of ASR as a function of time, yielding a regression coefficient (β), from which EAPC and 95% confidence intervals (95% CI) were computed using the equation:

$$EAPC=\left(e^{\beta }-1\right)\times 100$$

Additionally, percentage change was employed to quantify the relative shift in prevalence, incidence, and YLDs between 1990 and 2021, calculated as:

$$Percentage\;Change=\frac{{Value}_{2021}-{Value}_{1990}}{{Value}_{1990}}\times 100$$

### Association between socio-demographic index (SDI) and ASR

To explore the relationship between socioeconomic development and the burden of G6PD deficiency, we incorporated the Socio-Demographic Index (SDI) from the GBD 2021 framework. The SDI is a composite metric integrating three key indicators: Total Fertility Rate (TFR) under age 25; Mean years of education in individuals aged 15+; Lag-distributed income (LDI) *per capita*.

Data on gross domestic product (GDP), educational attainment, and fertility rates were extracted. Outliers and missing data were addressed through multiple imputation and sensitivity analyses, ensuring the reliability of the findings and minimizing potential biases. Principal component analysis (PCA) was applied to generate standardized SDI scores, which were subsequently classified into five tiers (High SDI, High-Middle SDI, Middle SDI, Low-Middle SDI, and Low SDI), reflecting varying levels of socioeconomic progress and health outcomes. SDI values range from 0 to 1, with 0 representing the lowest and one the highest level of development. SDI data for individual countries and regions were sourced from the IHME database (https://ghdx.healthdata.org/record/global-burden-disease-study-2021-gbd-2021-socio-demographic-index-sdi-1950%E2%80%932021). The association between SDI and ASR was evaluated using Spearman’s rank correlation analysis, while potential nonlinear trends were explored through a Locally Weighted Scatterplot Smoothing (LOESS) regression model.

### Statistical analysis

All statistical analyses were conducted using R software (version 4.3.3). Spearman’s rank correlation was employed to examine associations between SDI and G6PD burden, while LOESS regression was used to visualize non-linear patterns. Age-standardized trends were estimated using linear regression models. Data processing and plotting relied on standard epidemiological and visualization packages.

## Results

### Global prevalence, incidence, and years lived with disability (YLDs)

The number of prevalent cases and YLDs generally increased over the study period, although age-standardized YLD rates (ASYR) remained relatively stable. In contrast, incidence counts rose from 1990 to 2009, fluctuated between 2010 and 2017, and gradually declined from 2018 onward. Age-standardized YLD rates (ASYR) exhibited a steady decline since 1990. Meanwhile, both age-standardized incidence rates (ASIR) and age-standardized prevalence rates (ASPR) demonstrated an “N”-shaped pattern—rising between 1990 and 2006, decreasing from 2007 to 2015, and increasing again from 2016 to 2021. These inflection points may align with advances in diagnostics and shifts in screening policies (Supplementary Figure S1). In 2021, the estimated global prevalence of glucose-6-phosphate dehydrogenase (G6PD) deficiency was 443,326,869 (95% Uncertainty Interval [UI]: 408,259,369–482,326,895), reflecting an 80.17% increase compared to 1990 (Table 1). The age-standardized prevalence rate (ASPR) increased from 4542.59 per 100,000 (95% UI: 4213.51–4900.11) in 1990 to 5667.69 per 100,000 (95% UI: 5216.53–6171.34) in 2021, with an estimated annual percentage change (EAPC) of 0.46 (95% Confidence Interval [CI]: 0.29–0.64). In 2021, the global incidence of G6PD deficiency was 8,548,772 cases (95% UI: 7,734,372–9,462,072), an 18.54% increase compared to 1990 (Table 1). The age-standardized incidence rate (ASIR) rose to 138.19 per 100,000 (95% CI: 125.02–152.95), with an EAPC of 0.34 (95% CI: 0.15–0.52). The global YLD burden increased by 32.87%, rising from 7,211,453 person-years (95% UI: 6,629,823–7,878,123) in 1990 to 343,355 person-years (95% UI: 238,124–481,158) in 2021. The age-standardized YLD rate (ASYR) exhibited minimal variation over time, with an EAPC of 0.08 (95% CI: −0.16–0.33).

**TABLE 1:** The prevalence number and rate of G6PD in 1990 and 2021 across 21 regions and global, and the trends from 1990 to 2021.

Index	All ages number			Age-standard rate per 100,000 population		
	1990 (95% UI)	2021 (95% UI)	Percentage change,%	1990 (95% UI)	2021 (95% UI)	EAPC (95% CI)
Prevalence	246,062,082 (228,165,316, 265,541,550)	443,326,869 (408,259,369, 482,326,895)	80.17	4542.59 (4213.51, 4900.11)	5667.69 (5216.53, 6171.34)	0.46 (0.29, 0.64)
Incidence	7,211,453 (6,629,823, 7,878,123)	8,548,772 (7,734,372, 9,462,072)	18.54	112.54 (103.46, 122.94)	138.19 (125.02, 152.95)	0.34 (0.15, 0.52)
YLLs	258,412 (194,101, 342,687)	343,355 (238,124, 481,158)	32.87	0.39 (0.26, 0.57)	0.45 (0.30, 0.66)	0.08 (−0.16, 0.33)

### Regional disparities in G6PD deficiency burden

The highest G6PD deficiency burden in 2021 was observed in South Asia, which had the greatest prevalence (138,159,940 cases, 95% UI: 132,043,487–143,819,174), incidence (2,331,667 cases, 95% UI: 2,226,853–2,428,856), and YLD count (14616.19, 95% UI: 9630.7–21274.78) (Supplementary Table S1). The most substantial percentage increase in burden between 1990 and 2021 occurred in Andean Latin America, with a 291.96% increase in prevalence, 140.19% increase in incidence, and 104.73% increase in YLDs (Supplementary Table S1). In contrast, Central Europe and Eastern Europe exhibited a decline in prevalence, incidence, and YLDs over the same period (Supplementary Table S1).

The highest ASRs in 2021 were recorded in Central Sub-Saharan Africa (Table 2). More than 50% of regions experienced declining ASRs between 1990 and 2021 (EAPC <0), with Central Latin America and Southeast Asia exhibiting the most substantial reductions (Table 2). Conversely, Andean Latin America and the Caribbean demonstrated increasing ASRs, with the highest growth observed in Andean Latin America (Table 2).

**TABLE 2:** The prevalence, incidence and YLDs rate of G6PD in 1990 and 2021 across 21 regions, and the trends from 1990 to 2021.

Region	Prevalence			Incidence			YLDs		
	1990 (95% UI)	2021 (95% UI)	EAPC (95% CI)	1990 (95% UI)	2021 (95% UI)	EAPC (95% CI)	1990 (95% UI)	2021 (95% UI)	EAPC (95% CI)
Andean Latin America	1637.58 (1323.57, 2013.19)	3682.76 (2976.5, 4542.08)	1.90 (1.09, 2.71)	34.19 (27.63, 42.02)	77.77 (62.88, 95.94)	1.93 (1.12, 2.74)	2.71 (1.79, 3.85)	3.03 (2.06, 4.25)	0.13 (−0.70, 0.97)
Australasia	965.06 (774.32, 1196.24)	1469 (1211.17, 1739.38)	−0.04 (−0.55, 0.47)	19.85 (15.95, 24.57)	30.8 (25.36, 36.47)	0.14 (−0.34, 0.61)	0.03 (0.02, 0.04)	0.02 (0.01, 0.02)	−1.45 (−2.13, −0.76)
Caribbean	7366.84 (5958.53, 9080.73)	8543.37 (6926.7, 10522.83)	0.48 (0.46, 0.50)	186.45 (150.81, 230.06)	215.22 (174.46, 265.28)	0.43 (0.40, 0.46)	10.15 (6.84, 14.29)	12.31 (7.7, 19)	0.92 (0.81, 1.04)
Central Asia	1460.8 (1177.64, 1804.32)	1483.81 (1198.38, 1821.36)	0.09 (0.06, 0.11)	31.31 (25.22, 38.6)	32 (25.85, 39.29)	0.11 (0.08, 0.13)	0.86 (0.61, 1.18)	0.82 (0.61, 1.06)	−0.52 (−0.57, −0.47)
Central Europe	3129.26 (2713.92, 3628.92)	3391.09 (2946.18, 3928.17)	−0.20 (−0.52, 0.13)	67.23 (58.47, 77.73)	71.53 (62.14, 82.99)	−0.31 (−0.64, 0.03)	0.66 (0.46, 0.88)	0.23 (0.17, 0.3)	−2.12 (−2.46, −1.78)
Central Latin America	3790.5 (3340.83, 4299.57)	2732.74 (2350.99, 3201.18)	−1.11 (−1.30, −0.92)	78.26 (68.89, 88.82)	56.09 (48.06, 65.94)	−1.14 (−1.33, −0.95)	1.12 (0.79, 1.48)	0.75 (0.5, 1.1)	−1.80 (−1.92, −1.67)
Central Sub-Saharan Africa	13067.4 (10588.94, 16117.88)	12883.85 (10408.84, 15902.91)	−0.09 (−0.12, −0.06)	278.69 (225.77, 343.39)	270.15 (218.1, 333.21)	−0.14 (−0.17, −0.11)	28.63 (7.22, 90.06)	33.76 (7.86, 110.8)	−1.00 (−1.16, −0.84)
East Asia	3399.81 (3296.78, 3511.09)	4629.34 (4468.86, 4789.66)	0.92 (0.45, 1.40)	72.83 (70.62, 75.22)	96.14 (92.75, 99.54)	0.72 (0.27, 1.17)	4.97 (3.57, 7.08)	2.99 (2.24, 3.85)	−1.86 (−2.38, −1.34)
Eastern Europe	1987.97 (1891.03, 2073.79)	2054.18 (1962.73, 2142.42)	−0.04 (−0.13, 0.05)	42.81 (40.68, 44.72)	44.34 (42.36, 46.25)	−0.04 (−0.13, 0.06)	0.04 (0.03, 0.04)	0.03 (0.02, 0.03)	−1.39 (−1.60, −1.17)
Eastern Sub-Saharan Africa	9988.6 (8812.64, 11372.72)	8123.2 (7019.9, 9410.64)	−0.69 (−0.85, −0.53)	212.52 (187.27, 242.31)	175.96 (150.67, 205.73)	−0.62 (−0.78, −0.45)	16.29 (10.91, 25.86)	11.72 (7.17, 20.9)	−1.18 (−1.36, −1.00)
High-income Asia Pacific	542.01 (485.23, 606.93)	623.71 (550.44, 705.87)	0.19 (0.07, 0.31)	12.58 (11.03, 14.36)	12.41 (11.09, 13.91)	−0.29 (−0.41, −0.17)	1.13 (0.63, 1.68)	0.28 (0.17, 0.39)	−1.94 (−2.15, −1.72)
High-income North America	4877.72 (4698.88, 5061.75)	4187.07 (3990.99, 4352.81)	−0.45 (−1.32, 0.42)	103.66 (99.94, 107.5)	87.92 (83.9, 91.35)	−0.48 (−1.36, 0.42)	0.26 (0.24, 0.27)	0.01 (0.01, 0.01)	−0.56 (−1.53, 0.41)
North Africa and Middle East	6061.3 (5462.19, 6776.82)	5979.38 (5044.26, 7150.01)	0.34 (0.22, 0.46)	125.04 (111.59, 141.32)	116.37 (96.95, 140.73)	0.18 (0.05, 0.32)	7.06 (5.18, 9.68)	3.41 (2.34, 4.74)	−0.8 (−0.92, −0.69)
Oceania	6211.28 (5627.94, 6827.59)	5452.55 (4421.77, 6755.19)	−0.28 (−0.43, −0.14)	135.36 (122.95, 148.75)	116.9 (94.78, 144.85)	−0.35 (−0.5, −0.21)	8.5 (4.82, 13.45)	6.85 (4.4, 10.83)	−0.52 (−0.66, −0.39)
South Asia	5047.91 (4791.39, 5323.82)	7482.42 (7150.32, 7790.05)	0.10 (−0.42, 0.63)	100.54 (94.43, 107.23)	154.02 (147.09, 160.44)	0.20 (−0.32, 0.72)	3.33 (1.39, 5.12)	3.75 (1.6, 5.88)	−0.40 (−0.90, 0.10)
Southeast Asia	6147.24 (5642.79, 6736.34)	4435.14 (3958.98, 4988.87)	−0.97 (−1.16, −0.77)	128.14 (117.05, 141.29)	93.65 (83.79, 105.22)	−0.92 (−1.11, −0.73)	10.92 (8.41, 13.61)	6.44 (4.83, 8.34)	−2.12 (−2.33, −1.91)
Southern Latin America	1691.97 (1364.98, 2100.28)	1815.51 (1464.83, 2240.37)	−0.25 (−0.43, −0.07)	35.33 (28.49, 43.87)	38.15 (30.78, 47.03)	−0.23 (−0.41, −0.05)	0.02 (0.02, 0.03)	0.01 (0, 0.01)	−1.69 (−1.94, −1.44)
Southern Sub-Saharan Africa	8658.53 (8077.87, 9298.17)	8230.33 (7654.87, 8839.31)	−0.18 (−0.23, −0.13)	186.59 (171.51, 203.29)	176.16 (160.3, 193.77)	−0.21 (−0.27, −0.14)	14.53 (11.18, 18.29)	17.9 (13.3, 23.4)	−0.60 (−0.67, −0.53)
Tropical Latin America	2427.93 (2348.93, 2507.37)	3216.76 (3100.41, 3327.73)	1.45 (1.04, 1.87)	52 (50.25, 53.72)	70.66 (68.13, 73.09)	1.55 (1.11, 2.00)	0.09 (0.07, 0.11)	0.12 (0.08, 0.17)	−0.01 (−0.35, 0.33)
Western Europe	2483.11 (2219.33, 2774.39)	2570.31 (2263.17, 2913.16)	0.01 (−0.18, 0.20)	50.92 (45.37, 57.09)	52.76 (46.14, 60.4)	−0.01 (−0.19, 0.17)	0.04 (0.03, 0.04)	0.02 (0.02, 0.02)	−1.18 (−1.50, −0.86)
Western Sub-Saharan Africa	10954.23 (10185.21, 11735.93)	10087.71 (9182.3, 11107.93)	0.06 (−0.07, 0.19)	231.72 (215.13, 248.65)	218.81 (199.44, 240.78)	0.15 (0.01, 0.29)	18.87 (11.66, 28.65)	18.38 (10.03, 28.59)	−0.13 (−0.23, −0.03)

### National prevalence, incidence and YLDs

**FIGURE 1:**
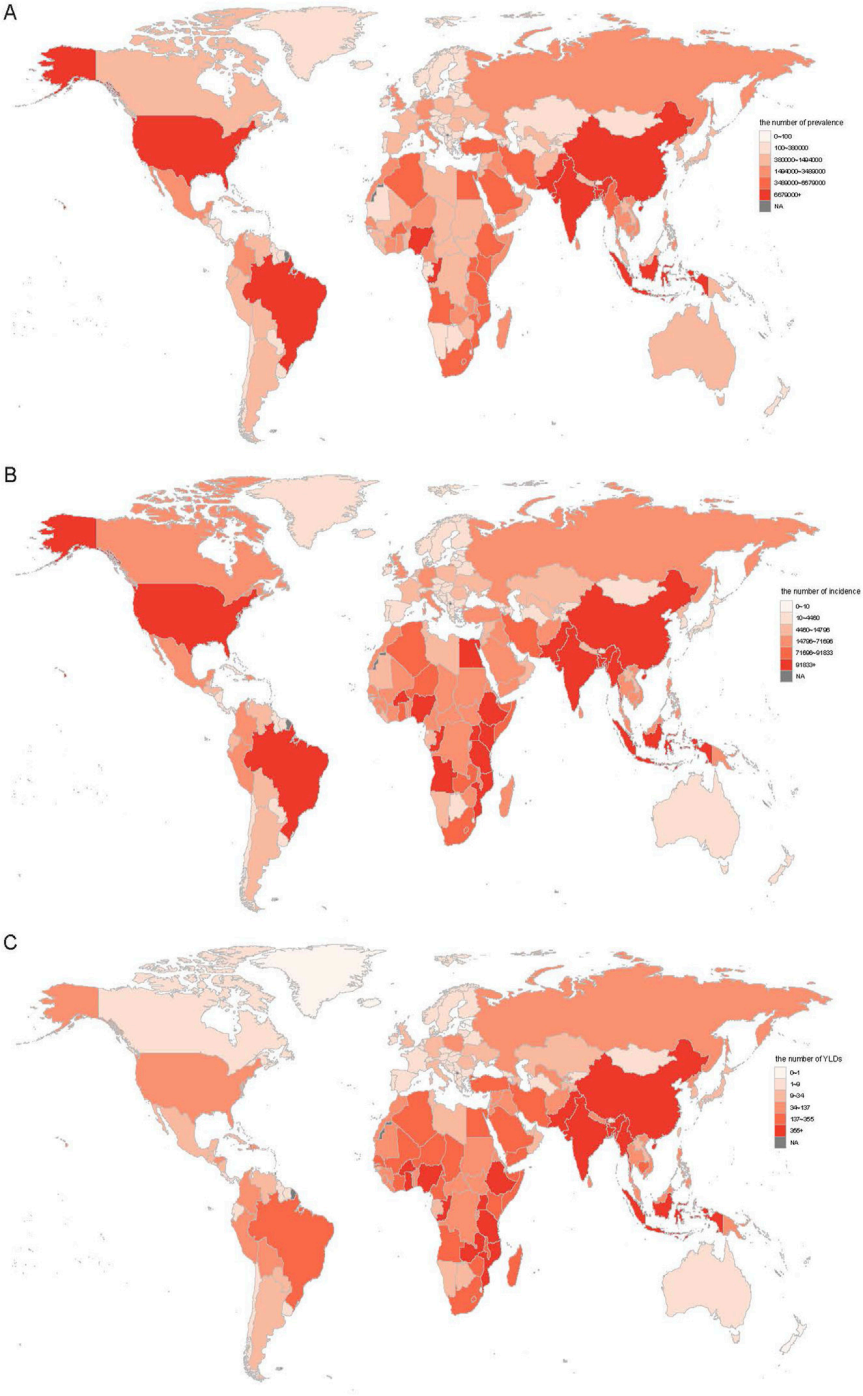
The all age prevalence **(A)**, incidence **(B)**, and YLDs **(C)** number for G6PD deficiency in 2021 across 204 countries. YLDs, years lived with disability.

In 2021, the largest G6PD deficiency burden was concentrated in India, China, and Nigeria, with India ranking highest in prevalence (115,096,664 cases, 95% UI: 111,191,436–118,670,315), incidence (1,860,560 cases, 95% UI: 1,796,964–1,917,769), and YLDs (12,143 person-years, 95% UI: 7,939–17,834) (Figure 1; Supplementary Table S2). Compared to 1990, approximately 86.3% (*n* = 176) of the 204 countries exhibited an increase in prevalence, while around 61.3% (*n* = 125) displayed a decline in incidence. The overlap between these categories (*n* = 97) arises from countries that showed increased prevalence but declining or stable incidence, indicating improvements in early detection without a corresponding rise in new cases.

In 2021, Gabon recorded the highest ASPR (16525.87 per 100,000, 95% UI: 13393.08–20374.18) and ASIR (351.18 per 100,000, 95% UI: 84.6–432.57), whereas Burkina Faso had the highest ASYR (1.89 per 100,000, 95% UI: 1.14–2.99) (Supplementary Table S3). Conversely, Japan exhibited the lowest ASPR (409.97 per 100,000, 95% UI: 392.64–426.78), ASIR (8.33 per 100,000, 95% UI: 7.97–8.67), and ASYR (0.01 per 100,000, 95% UI: 0–0.01) (Supplementary Table S3). New Zealand demonstrated the highest increases in ASPR (EAPC: 2.78, 95% CI: 0.95–4.64), ASIR (EAPC: 2.75, 95% CI: 0.98–4.55), and ASYR (EAPC: 1.45, 95% CI: −0.35–3.29), whereas Brunei exhibited the largest reductions in ASPR (EAPC: −4.37, 95% CI: −5.46 to −3.28) and ASIR (EAPC: −4.19, 95% CI: −5.33 to −3.03) (Supplementary Table S3). Notably, approximately 90% of countries experienced a decline in ASYR (EAPC <0, Supplementary Table S3).

### Burden of G6PD deficiency based on SDI

**FIGURE 2:**
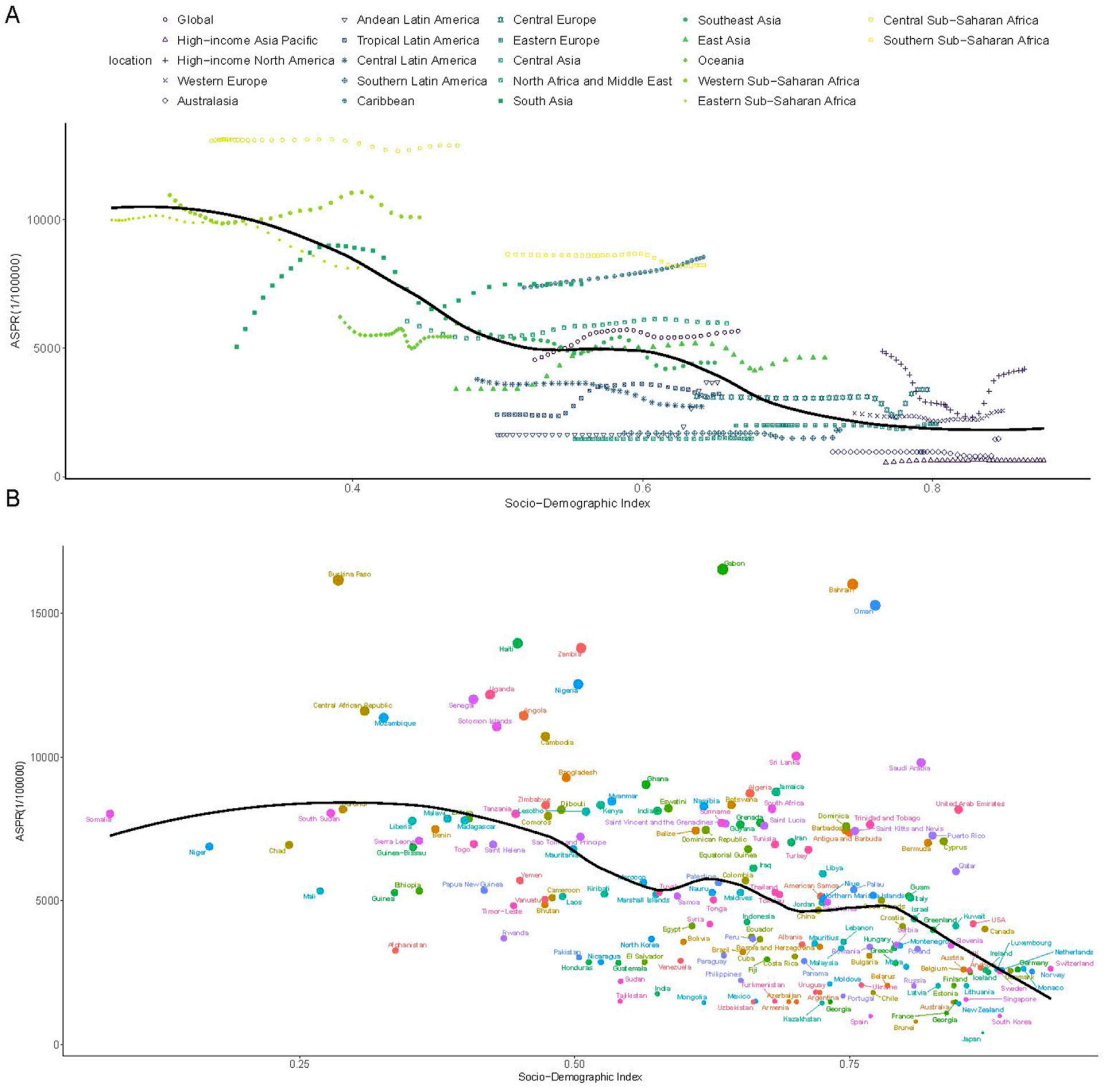
The association between the SDI and ASPR of G6PD deficiency. **(A)** The association between the SDI and ASPR in 21 regions and global according from 1990 to 2021; **(B)** The association between the SDI and ASPR in 204 countries in 2021. The black line was the expected values based on the SDI and disease rates. SDI, socio-demographic index; ASPR, age-standardized prevalence rate.

Between 1990 and 2021, ASPR demonstrated an overall negative correlation with SDI (Figure 2A). However, a positive correlation was observed in Central Europe, Eastern Europe, South Asia, Western Sub-Saharan Africa, and Southern Sub-Saharan Africa, where ASPR increased with rising SDI levels (Figure 2A). ASPR was also higher than expected based on SDI in regions such as Central Sub-Saharan Africa, Southern Sub-Saharan Africa, the Caribbean, Western Europe, and High-Income North America (Figure 2A). At the national level, ASPR exhibited a negative correlation with SDI in 2021 (Figure 2B). Countries such as Gabon, Burkina Faso, Bahrain, and Oman displayed ASPR values exceeding expected levels (Figure 2B). Trends in ASIR and ASYR mirrored those of ASPR (Supplementary Figures S2, S3).

### Burden of G6PD deficiency based on age and sex

**FIGURE 3:**
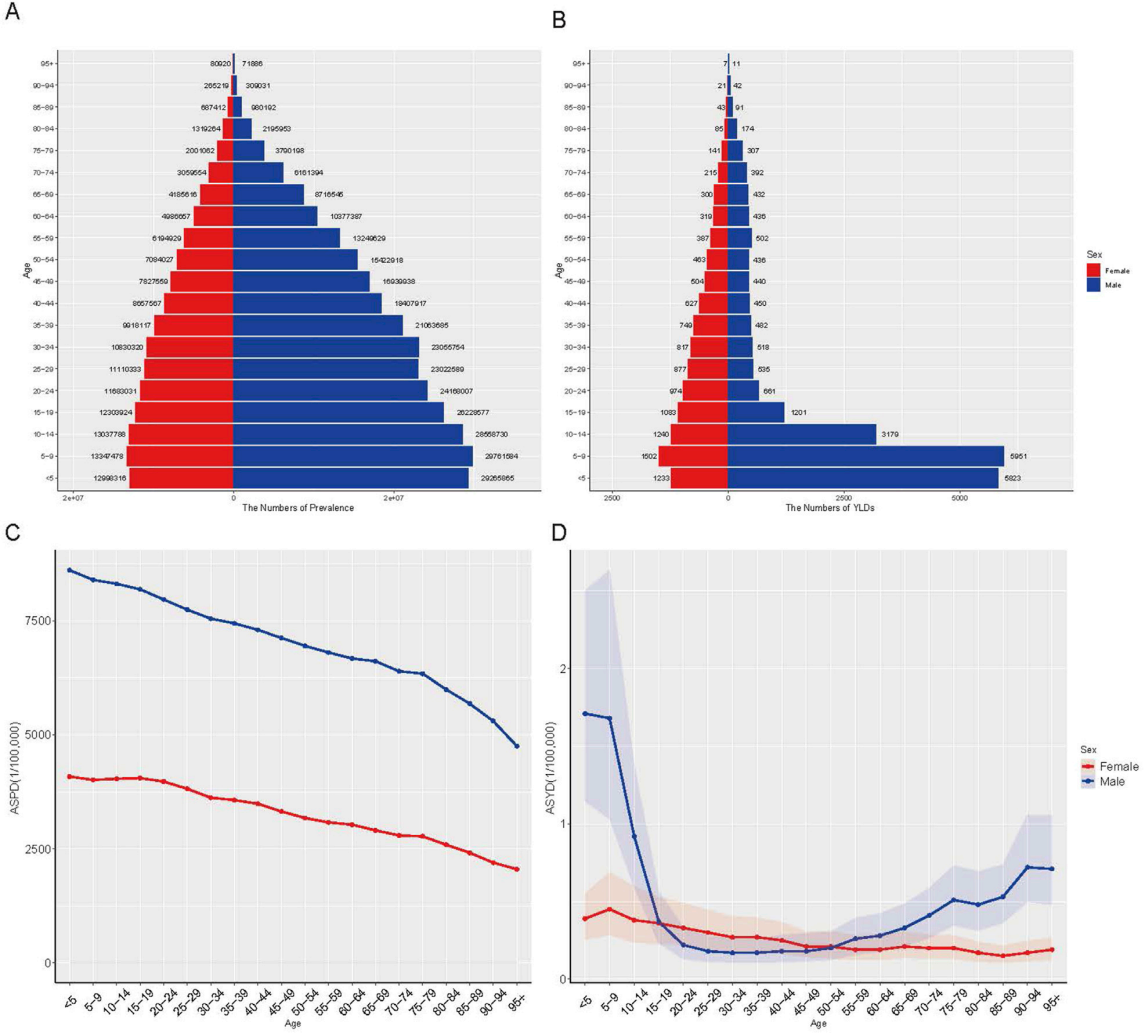
The global disease burden of G6PD deficiency by age and sex in 2021. **(A)** The number of prevalence; **(B)** The number of YLDs; **(C)** ASPR; **(D)** ASYR. YLDs, years lived with disability; ASPR, age-standardized prevalence rate; ASYR, age-standardized YLDs rate.

In 2021, prevalence and ASPR declined with increasing age from 5 to 9 years onward, with males exhibiting higher prevalence and ASPR across all age groups (Figures 3A,C). Among females, YLD count and ASYR decreased with age progression from 5 to 9 years onward (Figures 3B,D). Among males, YLD count and ASYR followed a U-shaped trend, declining initially before rising in older age groups. Males exhibited higher YLDs and ASYR than females before age 19 and after age 55, but lower values between ages 20 and 54 (Figures 3B,D). Due to the absence of age-stratified incidence data in GBD 2021, incidence and ASIR by age and sex were not analyzed.

## Discussion

This study presents the most comprehensive assessment of G6PD deficiency burden to date, utilizing GBD 2021 data to track trends over a 32-year period across 204 countries and territories. The findings underscore a substantial rise in global prevalence, with 443.3 million cases reported in 2021, marking an 80.17% increase since 1990. Global prevalence of G6PD deficiency was estimated at approximately 246 million in 1990 and increased to approximately 443 million in 2021, marking an 80.17% rise over the 31-year period. This significant growth may reflect multiple interrelated factors, including demographic shifts, improved diagnostic capabilities, expanded newborn screening, and increased global migration from high-prevalence regions (Cappellini and Fiorelli, 2008; Tabatabaei et al., 2015; Xuan-Rong Koh et al., 2023).

Diagnostic efforts have evolved significantly, with methodologies transitioning from manual, laboratory-intensive assays to rapid, portable biosensors such as CareStart™, BinaxNOW^®^, and the STANDARD™ G6PD test, substantially enhancing case detection capacities in resource-limited settings. These advances, coupled with integrated newborn screening programs in high-prevalence countries like India, Brazil, and Nigeria, have markedly reshaped the epidemiological landscape. While these advances have contributed to better disease recognition in some settings, they also highlight persistent disparities in healthcare access and disease management worldwide.

A key observation in this study is the heterogeneous geographical distribution of G6PD deficiency. As expected, South Asia and Sub-Saharan Africa remain the most heavily affected regions, where historical malaria exposure has maintained high allele frequencies (Cappellini and Fiorelli, 2008; Bancone et al., 2018). However, the fastest-growing burden was observed in Andean Latin America (+291.96%), a trend likely influenced by increased diagnostic efforts, improved healthcare infrastructure, and genetic admixture due to population migration (Awab et al., 2021). These findings emphasize the importance of region-specific screening and intervention strategies, as disease burden is no longer confined to traditionally endemic areas.

The association between SDI and G6PD burden provides crucial insights into the socioeconomic determinants of disease prevalence and healthcare accessibility. A negative correlation was observed between SDI and age-standardized prevalence rates (ASPR), indicating a disproportionately higher burden in lower-SDI regions (Tang et al., 2024). This aligns with existing evidence that low-resource settings often lack access to systematic screening, public health education, and neonatal monitoring programs, leading to underdiagnosis and an increased risk of severe hemolytic complications (Watson et al., 2019). Conversely, high-SDI countries exhibited a higher-than-expected prevalence, possibly due to enhanced diagnostic sensitivity and rising immigration from high-prevalence regions (Watson et al., 2019). These findings highlight the dual challenge of expanding screening in low-SDI settings while refining diagnostic protocols in high-SDI regions to prevent overestimation of disease burden. Although a clear association between G6PD deficiency prevalence and SDI was observed, the descriptive nature of this ecological analysis precludes any causal interpretation.

The age- and sex-specific burden of G6PD deficiency further refines our understanding of its clinical and epidemiological impact. As anticipated, males exhibited a significantly higher prevalence than females due to the X-linked inheritance pattern, resulting in more severe clinical manifestations among hemizygous males (Cappellini and Fiorelli, 2008; Dore et al., 2021; Zhou et al., 2024). A concerning finding is the high burden of neonatal hyperbilirubinemia, which remains a leading cause of kernicterus and long-term neurodevelopmental impairments in affected infants (Taylor et al., 2023). Despite advances in neonatal care, disparities persist, particularly in low-SDI countries where access to phototherapy, bilirubin screening, and exchange transfusions remains limited (Arain and Bhutani, 2014; Goyal et al., 2015; Pao et al., 2005). This underscores the urgent need for universal neonatal screening programs, particularly in high-burden regions where undiagnosed cases contribute to preventable morbidity and mortality.

Beyond infancy, the burden of G6PD deficiency follows a U-shaped trend in years lived with disability (YLDs), peaking in early childhood and again in older age groups (Bancone et al., 2018). This suggests that while acute hemolytic episodes predominate in younger populations, older individuals with chronic hemolysis may develop cumulative complications over time (Kwiatkowski, 2005; Galatas et al., 2017). Aging populations with G6PD deficiency may experience an increased risk of chronic anemia, oxidative stress-related comorbidities, and a heightened susceptibility to infections or drug-induced hemolysis, necessitating lifelong disease monitoring and adapted management strategies (Malaria Genomic Epidemiology Network, 2014; Cappellini and Fiorelli, 2008; Kwiatkowski, 2005).

This study leverages the comprehensive and standardized GBD 2021 database, which enables robust cross-national and longitudinal comparisons of G6PD deficiency burden. The inclusion of consistent case definitions and harmonized methodology across 204 countries and territories strengthens the credibility of global trends. However, several limitations warrant cautious interpretation. Variability in reporting quality across regions and the limited availability of subnational data may introduce inconsistencies. Furthermore, the observed rise in prevalence likely reflects demographic expansion and improved case detection, rather than a true increase in underlying disease risk. These factors underscore the importance of contextualizing results within local healthcare system capacities and diagnostic capabilities. From a public health perspective, the findings of this study underscore the urgent need for targeted interventions. Universal newborn screening for G6PD deficiency has proven effective in reducing morbidity, yet its implementation remains inconsistent across countries (Bancone et al., 2018; Pukrittayakamee et al., 2024; Kuupiel et al., 2020). Expanding affordable, point-of-care diagnostic testing is critical, particularly in low-income and high-prevalence settings where disease burden is highest (Galatas et al., 2017; Zobrist et al., 2021; LaRue et al., 2014). In addition, comprehensive patient education initiatives must be prioritized to mitigate avoidable hemolytic triggers, particularly in regions where awareness among healthcare providers and affected communities remains low (Kuupiel et al., 2020; Ciavarella et al., 2025). Another key challenge lies in the intersection between G6PD deficiency and antimalarial treatment policies. The widespread use of primaquine and tafenoquine for malaria eradication necessitates careful risk stratification, as these drugs are strong oxidants capable of inducing severe hemolysis in G6PD-deficient individuals (Sulistyaningrum et al., 2020; Mukherjee et al., 2015). Balancing malaria control efforts with patient safety will require expanded pre-treatment screening, alternative drug regimens, and the development of G6PD-safe antimalarial therapies (Ciavarella et al., 2025; Sulistyaningrum et al., 2020; Watson et al., 2017; Chu et al., 2018).

To further enhance global efforts against G6PD deficiency, future research should focus on integrating genomic epidemiology into routine public health surveillance, refining diagnostic algorithms to reduce false-positive rates, and exploring genotype-phenotype correlations to optimize individual patient management. These directions will help transition from descriptive epidemiology to precision medicine approaches, ensuring better-targeted interventions and equitable health outcomes across diverse settings.

In conclusion, this study provides critical epidemiological insights into the evolving global burden of G6PD deficiency, emphasizing the need for region-specific screening policies, healthcare accessibility improvements, and refined clinical management strategies. Addressing these challenges through precision medicine, global health collaborations, and expanded neonatal care programs will be essential in mitigating the long-term impact of this disorder and ensuring equitable healthcare for affected populations worldwide.

## Data Availability

The datasets presented in this study can be found in online repositories. The names of the repository/repositories and accession number(s) can be found in the article/Supplementary Material.
