# Supplementary material for: Global, regional, and national burden of glucose-6-phosphate dehydrogenase (G6PD) deficiency from 1990 to 2021: a systematic analysis of the global burden of disease study 2021
Source: Front Genet. 2025 May 23;16:1593728. doi: 10.3389/fgene.2025.1593728 (PMC12141281; doi:10.3389/fgene.2025.1593728)
Supplement: Supplementary file 1 [file DataSheet1.pdf]

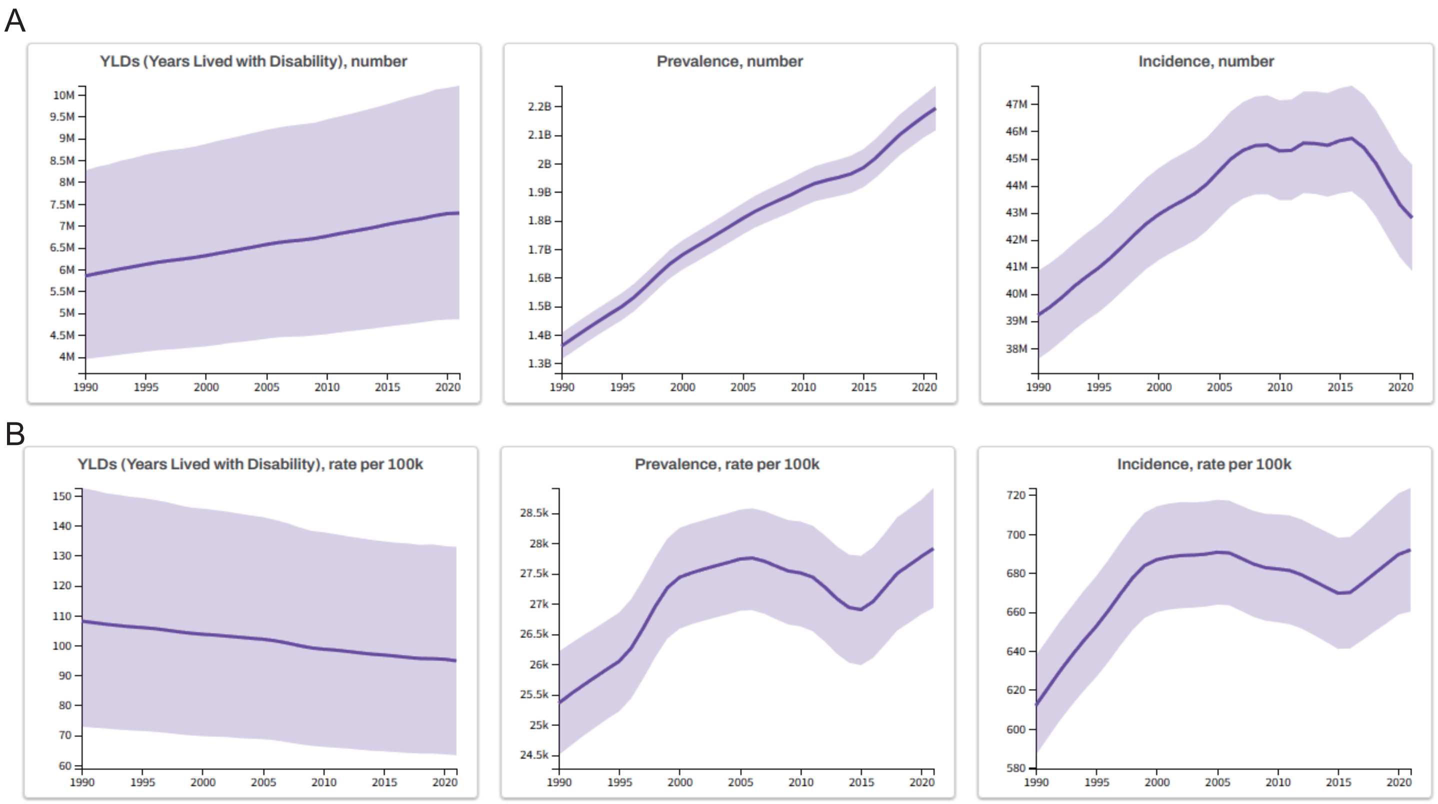

Supplementary Figure S1 Globally temporal trend of G6PD deficiency from 1990 to 2019. (A) The temporal trend for number of YLDs, prevalence and incidence from 1990 to 2021; (B) The temporal trend for age-standardized rates of YLDs, prevalence and incidence from 1990 to 2021.

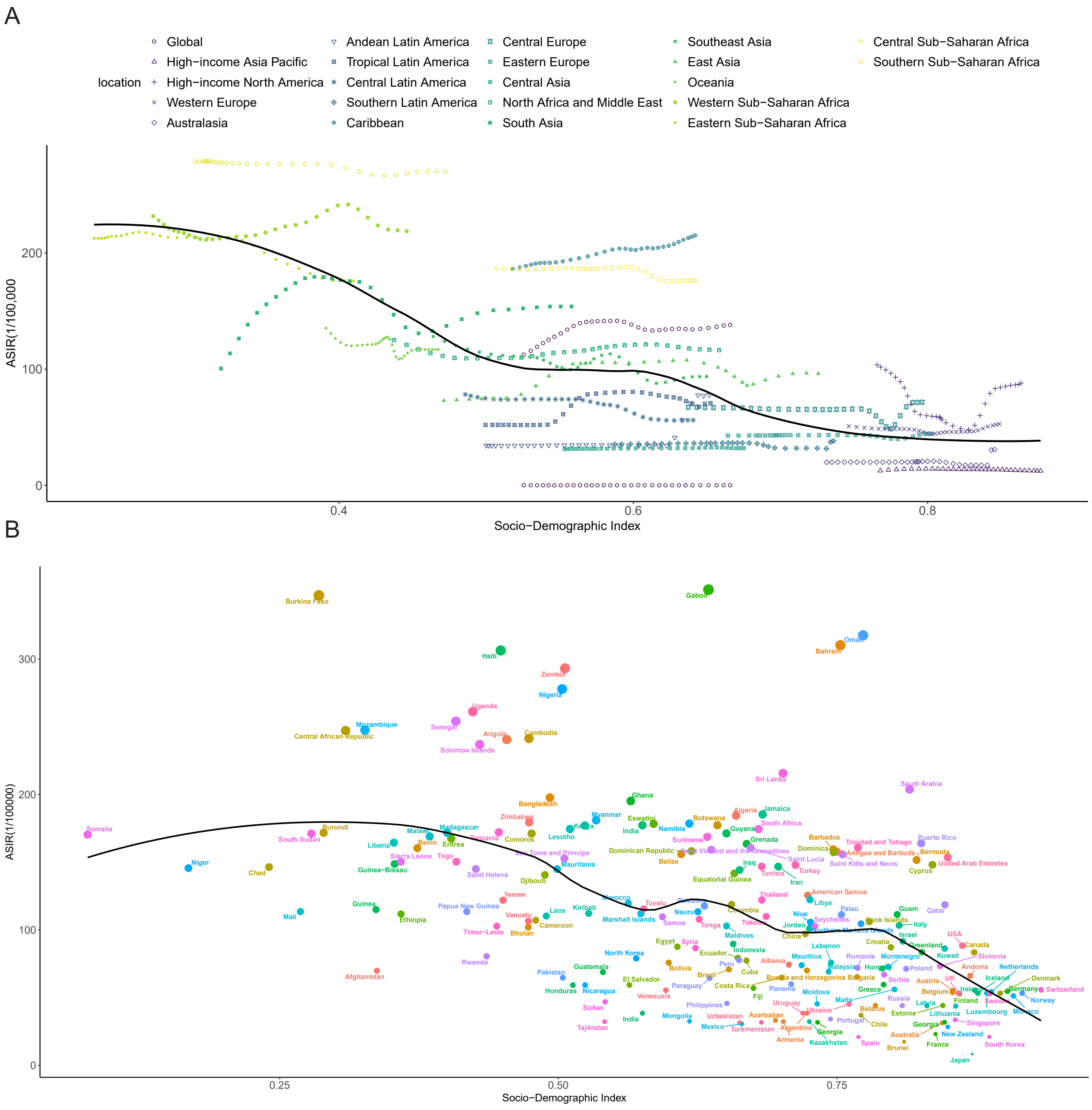

Supplementary Figure S2 The association between the SDI and ASIR of G6PD deficiency. (A) The association between the SDI and ASIR in 21 regions and global according from 1990 to 2021; (B) The association between the SDI and ASIR in 204 countries in 2021. The black line was the expected values based on the SDI and disease rates. SDI, socio-demographic index; ASIR, age-standardized incidence rate.

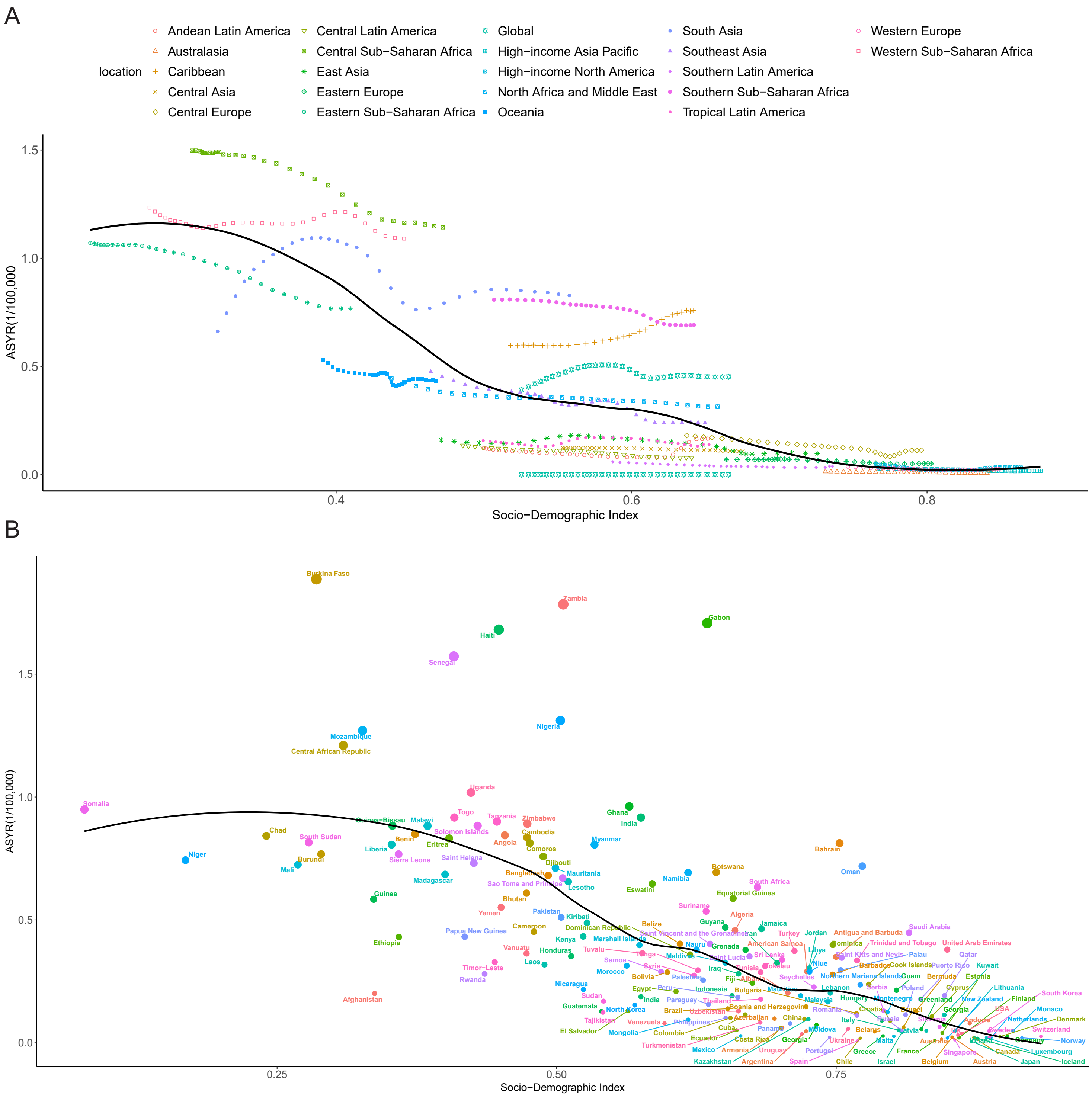

Supplementary Figure S3 The association between the SDI and ASYR of G6PD deficiency. (A) The association between the SDI and ASYR in 21 regions and global according from 1990 to 2021; (B) The association between the SDI and ASYR in 204 countries in 2021. The black line was the expected values based on the SDI and disease rates. SDI, socio-demographic index; ASYR, age-standardized YLDs rate.
